# South-to-North Water Diversion stabilizing Beijing’s groundwater levels

**DOI:** 10.1038/s41467-020-17428-6

**Published:** 2020-07-21

**Authors:** Di Long, Wenting Yang, Bridget R. Scanlon, Jianshi Zhao, Dagen Liu, Peter Burek, Yun Pan, Liangzhi You, Yoshihide Wada

**Affiliations:** 1 State Key Laboratory of Hydroscience and Engineering, Department of Hydraulic Engineering, Tsinghua University, Beijing, 100084 China; 2 Bureau of Economic Geology, Jackson School of Geosciences, The University of Texas at Austin, Austin, TX 78758 USA; 3 Beijing Water Authority, Beijing, 100038 China; 4 International Institute for Applied Systems Analysis, 2361 Laxenburg, Austria; 5 College of Resources Environment and Tourism, Capital Normal University, Beijing, 100048 China; 6 Macro Agriculture Research Institute (MARI), College of Economics and Management, Huazhong Agricultural University, Wuhan, 430070 China; 7 International Food Policy Research Institute (IFPRI), Washington, DC 20005 USA

**Keywords:** Hydrology, Water resources

## Abstract

Groundwater (GW) overexploitation is a critical issue in North China with large GW level declines resulting in urban water scarcity, unsustainable agricultural production, and adverse ecological impacts. One approach to addressing GW depletion was to transport water from the humid south. However, impacts of water diversion on GW remained largely unknown. Here, we show impacts of the central South-to-North Water Diversion on GW storage recovery in Beijing within the context of climate variability and other policies. Water diverted to Beijing reduces cumulative GW depletion by ~3.6 km^3^, accounting for 40% of total GW storage recovery during 2006–2018. Increased precipitation contributes similar volumes to GW storage recovery of ~2.7 km^3^ (30%) along with policies on reduced irrigation (~2.8 km^3^, 30%). This recovery is projected to continue in the coming decade. Engineering approaches, such as water diversions, will increasingly be required to move towards sustainable water management.

## Abstract (web summary)

The authors here address water sustainability in the greater area of Beijing, China. Specifically, the positive effects towards Beijing groundwater levels via water diversion from the Yangtze River to the North are shown.

## Introduction

Groundwater (GW) is a critical resource globally, especially in semiarid regions, accounting for ~26% of global water withdrawals and ~40% of irrigation water consumption^1,2^. About 70% of GW abstraction contributes to the agricultural sector globally^1^. Overexploitation of GW due to population growth, higher gross domestic product and related dietary changes, and climate change/variability could significantly impact water and food security^3–6^. Many studies have reported groundwater depletion (GWD) in major aquifers globally^7–11^, such as those in Northwest India^12^, U.S. California’s Central Valley^13^, U.S. High Plains^14^, Middle East^15^, and the North China Plain (NCP)^16–18^. GWD in the NCP is among the highest, with depletion of 3.5 km^3^ year^−1^ during 1983–1993 based on shallow groundwater level data^18^ and 8.3 km^3^ year^−1^ during 2003–2010 based on GRACE (Gravity Recovery And Climate Experiment) satellite observations, including mass changes of both shallow and deep aquifers, and marked depletion locally in Beijing within the NCP based on groundwater level data^16^ (Supplementary Table 1).

Water crises threaten many large cities globally. Mexico City experienced land subsidence up to 30 cm year^−1^ due to massive GW extraction for municipal and agricultural water use, decreasing water resources, damaging infrastructure, and increasing flood risk and water contamination^19^. Other cities, such as Melbourne, Jakarta, Chennai, Sao Paulo, and major cities in India, have also experienced severe water scarcity, and marked GWD globally has threatened water supply for municipal and agricultural use and resulted in land subsidence and drying-up of rivers and wetlands^3,12,20–25^. Due to intensive agricultural irrigation, rapid urbanization, and a relatively dry climate, the NCP and associated megacities, including China’s capital of Beijing with 21.5 million residents in 2018, have experienced severe water shortages over the past two decades. The mean GW depth in the Beijing Plain declined at a rate of ~0.7 m year^−1^ from 15 m below the ground surface in 2000 to 26 m in 2014^26^. Compounded by changing patterns of precipitation and GW recharge^27–30^, and increasing climate extremes, such as drought^31,32^ and heatwaves^33^, Beijing’s water supply was projected to worsen in coming years.

Increasing the resilience of water supplies for large cities to climate extremes and change, particularly severe droughts, is critical. Various approaches can be used to address water management to enhance resilience. Water storage reflects the balance between water inputs or supplies and outputs or demands. Historical storage depletion may have resulted from reduction in inputs (e.g., reduced recharge from lower precipitation or drought) or increased output (e.g., irrigation pumpage), or both. Different approaches can be applied to recover water storage, including increasing inputs and/or reducing outputs. Inputs can be increased by transporting water from more humid regions to arid regions (e.g., California and Arizona^34^) and developing alternative water sources (e.g., desalinating seawater as in Melbourne^35^ and Cape Town^36^). Outputs can be reduced by decreasing pumpage or recycling water. Conjunctive use of surface water (SW) and GW can also improve water management by using SW during wet periods and restricting GW use to drought periods. Managed aquifer recharge is also valuable for storing excess SW from wet periods in depleted aquifers for use during dry periods, evening out water supply variability^36^.

**Fig. 1:**
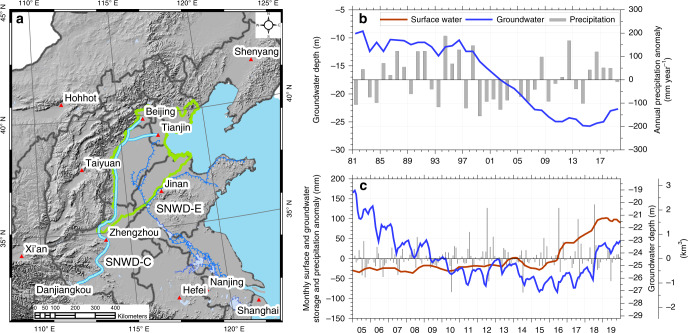
SNWD routes, water storage, and precipitation in Beijing. **a** Central (SNWD-C, light blue line) and eastern (SNWD-E, dark blue line) routes of the SNWD (South-to-North Water Diversion), capitals of provinces and Beijing and Tianjin Municipalities (red triangles) in North China, and the NCP (North China Plain, the green polygon). The western route is still being designed and not shown. **b** Mean GW depths at the end of each year and corresponding annual precipitation anomalies during 1981‒2019 (the first two numbers of the years are omitted in these figures for brevity), with a mean annual precipitation (MAP) of 540 mm year^−1^ for the period. **c** Monthly SW and GW storage anomalies (or GW depths, see the “Methods” section and Supplementary Fig. 1) and associated monthly precipitation anomalies during 2005‒2019. The climatology of monthly precipitation for calculating the precipitation anomalies was derived from the 2002‒2019 period.

GRACE satellites monitor changes in Earth’s gravity field that is controlled primarily by variations in total water storage^37,38^, showing that total water storage is decreasing or getting drier in North China whereas storage is increasing or getting wetter in South China^10,16^. One solution to ease water shortages in the NCP and Beijing would be to divert water from the humid south. The South-to-North Water Diversion (SNWD) was launched in 2002 and planned to transport ~45 km^3^ of water annually from the Yangtze River through three canal and pipeline systems (Supplementary Note 1). The objective of this study was to quantify the impacts of the central SNWD on groundwater storage (GWS) in Beijing. The central SNWD is projected to divert 9.5 km^3^ year^−1^, with the long-term goal of diverting 13 km^3^ year^−1^ expected to be realized in 2030, flowing from the Danjiangkou Reservoir (the water-source region) with a capacity of 29 km^3^ in the middle reach of the largest tributary (Han River) of the Yangtze River to more than 20 major cities, including Beijing (population 21.5 million) and Tianjin (population 15.6 million) municipalities and other cities in Henan and Hebei Provinces in North China (Fig. 1a).

Previous studies evaluated impacts of climate variability and water diversion on GWS over the Beijing Plain using GW flow models or coupled SW/GW models to simulate changes in GW levels under different climate and water diversion scenarios^39–41^. However, the effects of policies that restrict GW extraction on GWS were omitted. In addition, the lack of physical mechanisms for the GW recharge simulations (e.g., percolation and preferential flow) may result in erroneous evaluation for the impacts of climate variability on GWS. Contributions of climate variability, water diversion, and policies to enhance GW recovery for Beijing have not been quantified.

The novel aspect of this analysis was to quantify SNWD impacts within the context of the effects of climate extremes and water policies (e.g., reduction in irrigation pumpage) using physically based hydrologic modeling, and to project how these factors may influence GWS in the future. This study benefits from using multisource monitoring data on GW and water use of different sources and sectors as well as a sophisticated numerical model to: (1) assess various scenarios to isolate impacts of the central SNWD relative to climate variability and water policies and (2) to predict GWS changes under various precipitation and GW use scenarios. A flowchart showing the methodology of this study can be found in Supplementary Fig. 2. Results indicate that the diverted water to Beijing reduces GWD by ~3.7 km^3^ during 2006–2018, accounting for 40% of the total GWS recovery. Suppression of agricultural water use and increased precipitation reduce GWD by ~2.8 km^3^ (30%) and ~2.7 km^3^ (30%), respectively. GWS is project to continue to recover under various combinations of precipitation and water use scenarios for the incoming decade (2021–2030). The following “Results” section shows key analyses and results based on statistical and monitoring data (i.e., the first two subsections) and hydrologic modeling (i.e., the last two subsections).

## Results

### Groundwater storage depletion in Beijing

Beijing has less than 100 m^3^ per capita water resources within its administrative area, 1/20 of the national average, 1/80 of the global average, and far below the internationally recognized danger level of 1700 m^3^ and absolute scarcity of less than 500 m^3,42^. GW in Beijing has largely been depleted since a prolonged drought during 1999–2007 (Fig. 1b), which was amplified by increasing human water use with an average of 3.6 km^3^ year^−1^ between 2003 and 2018, in which 2.6 km^3^ year^−1^ was derived from renewable water resources (i.e., SW and GW that are naturally formed by precipitation)^26^. Prior to the SNWD, the discrepancy of ~1 km^3^ between water demand and supply was made up by GW withdrawal, depleting these reserves and resulting in long-term GWS decline of 17.5 ± 0.8 mm year^−1^ (~0.3 km^3^ year^−1^, *p* < 0.01) during 2005–2014 (Fig. 1c).

SW storage (SWS, all major reservoir water storage across the city) generally increased as a result of human water management to strategically store water for use in emergency situations, such as droughts. During 2005–2019, annual precipitation in Beijing was lowest in 2014 (443 mm year^−1^), particularly during July 2014 (79 mm, relative to mean July precipitation of 164 mm, Fig. 1c). SWS acted to buffer the effects of drought on human and natural water use, with a marked decline in SWS in 2014. Since the central SNWD operated in December 2014, SWS increased more than doubled (i.e., from 1.4 km^3^ in June 2015 to 3.3 km^3^ in December 2019).

The mean GW depth records indicate recovery during 2011–2012 prior to the SNWD and during 2015–2019 since the central SNWD started to operate (Fig. 1c). In particular, the mean GW depth in the Beijing Plain rose by 3 m over 5 years (25.7 m in December 2014 to 22.7 m in December 2019). GWS recovery can result from increased recharge related to higher precipitation and/or decreased GW withdrawals. The marked early recovery in GWS is attributed to elevated precipitation that increased from 552 mm year^−1^ (2011) to 708 mm year^−1^ (2012), the highest recorded annual precipitation during 2000–2019. GWS recovery since 2015 is compounded by both climate variability and human activity, including reduced pumpage and water diversions.

### Water supply and use in Beijing

**Fig. 2:**
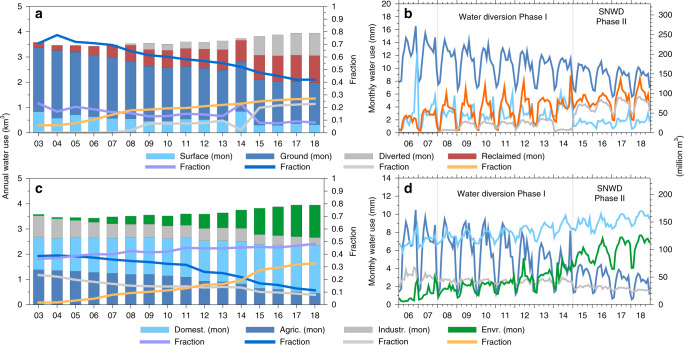
Water use in Beijing for 2003–2018. **a** Annual water use (bars) in terms of different water sources (km^3^) and annual fractions of total water use (lines), including SW, GW, diverted, and reclaimed water. **b** Monthly water use in terms of different water sources (mm in terms of water thickness or million m^3^ in terms of volume). **c** Annual water use (bars) in terms of different water use sectors (km^3^) and their annual fractions of total water use (lines), including domestic (i.e., households, service sector, and construction), agricultural, industrial, and environmental water use. **d** Monthly water use in terms of different water use sectors (mm in terms of water thickness or million m^3^ in terms of volume). (mon) refers to monthly in terms of line graphs in (**b**, **d**).

**Fig. 3:**
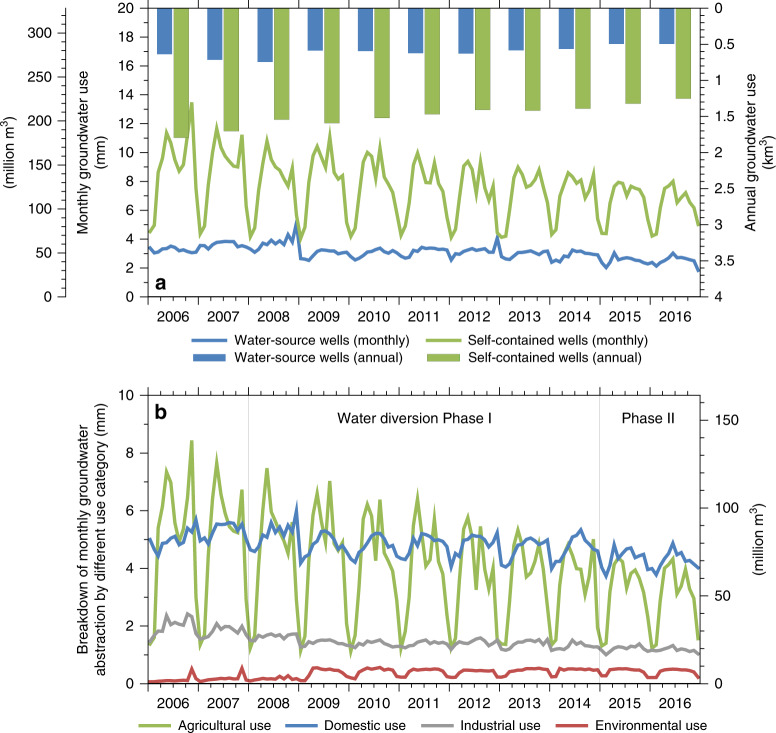
Groundwater use and its breakdown in Beijing for 2006–2016. **a** Monthly and annual GW use, including self-contained wells that mainly supply water for agricultural and domestic use, and water-source wells that mainly supply water for domestic and industrial use. **b** Breakdown of monthly GW abstraction by different use categories in Beijing during 2006‒2016, including agricultural, domestic, environmental, and industrial use.

Annual and monthly water use data from Beijing Water Resource Statistics Year Books and Beijing Water Authority (“Data availability” section) in terms of water source (e.g., GW and SW use) and sector (e.g., agricultural and domestic use) were examined to understand their interannual and seasonal variability (Figs. 2, 3). Understanding these variations is essential for assessing the causes for reductions in GW withdrawal and thereby formulation of future GW withdrawal scenarios. A total of 6.8 km^3^ of water has been diverted from 2008 to 2019 (in two phases, i.e., 1.6 km^3^ for 2008–2014 related to emergency water supply from major reservoirs in Hebei Province close to Beijing and 5.2 km^3^ for 2015–2019 related to the SNWD, Supplementary Note 2) to meet Beijing’s water demand.

GW withdrawals contributed to domestic (45%), agricultural (38%), industrial (14%), and environmental (3%) water use and have been steadily decreasing from 2.54 km^3^ (70% of total water use) in 2003 to 1.66 km^3^ in 2018 (42%) (Figs. 2a, b, 3a). The decline is attributed to two major factors: (1) increasing replacement of a portion of GW withdrawal with the SNWD for domestic use (Figs. 2, 3), e.g., the contribution of GW to domestic water use decreased from ~64% during 2006–2014 to ~48% during 2015–2016 after the SNWD, and (2) GW restrictions on agricultural water use by the government (Figs. 2c, d, 3), which declined from 1.38 km^3^ in 2003 (accounting for 40% of total water use) to 0.45 km^3^ in 2018 (11%). Because 80% of agricultural water use was sourced from GW, marked declines in irrigation decreased GW withdrawals.

Changes in sectoral water use have been driven by urbanization, changes in socioeconomic structure, and implementation of water-related policies. Both agricultural and industrial water use have decreased (Figs. 2c, d, 3b), alleviating pressure on GW withdrawal. In contrast, domestic and environmental water use increased, with domestic water use accounting for the largest share of total water use increasing up to 45% since 2011. Domestic water use, including water use from households, the service sector, and construction, increased to a certain degree (2016: 1.76 km^3^; 2017: 1.84 km^3^; 2018: 1.90 km^3^). The slight increase in domestic water use was driven primarily by increases in water use by households and the service sector as the social economy improved (e.g., increased incomes, residential areas, and living standards). However, the rate of increase in domestic water use slightly decreased from 0.08 km^3^ in 2017 to 0.06 km^3^ 2018, indicating that the domestic water use should not grow substantially, if the current policies on restricting the number of residents and transferring the functions of the capital to other regions continue to be implemented.

GW withdrawals (2006–2016) from self-contained wells (i.e., wells owned by some factories, mines, colleges, and farmers for their own use) vary by sector: agricultural (53%), domestic (31%), industrial (11%), and environmental use (5%) in Beijing. GW withdrawals from all self-contained wells (Fig. 3a) decreased by 30% from 1.79 km^3^ in 2006 to 1.25 km^3^ in 2016, mostly attributed to reduced agricultural water use during the entire study period and reduced GW withdrawals for domestic use during the water diversion (Fig. 3b). This is similar to the case of water-source wells (i.e., wells owned by waterworks that supply water through the public pipe network system), ~77% of which supply water for domestic use and ~21% for industrial use. GW withdrawals from water-source wells have declined by 22% from 0.64 km^3^ in 2006 to 0.50 km^3^ in 2016 due to substitution by diverted water (Fig. 3a). In terms of seasonality, GW from self-contained wells and for agricultural water use exhibit biannual peaks (Fig. 3), generally corresponding to irrigation periods for winter wheat (March–May) and summer maize (July–August).

Conjunctive use of SW and GW exemplifies a new water use structure in Beijing, contributing to more sustainable GW use. SW use has declined due to a strategy of increasing reservoir water storage for emergency use, as exemplified by the sharp decrease in stored SW to address the water supply crisis (i.e., increased SW use) during the summer drought in 2014 (Figs. 1c, 2a, b). The use of reclaimed wastewater has increased, contributing mostly to environmental water use (e.g., watering trees and grass and replenishing lakes and rivers). The government is improving the environment to meet citizens’ needs, increasing use of reclaimed water and allocating a portion of diverted water for environmental use in recent years. About 67% (~3.5 km^3^) of the total diverted water (5.2 km^3^ from 2015 through 2019) from the central SNWD was allocated for domestic and industrial use following treatment at waterworks. About 19% (~1 km^3^) was allocated for environmental use and the remaining 14% (~0.7 km^3^) was strategically stored in the Miyun Reservoir (Supplementary Fig. 3). More than 80% of agricultural water use was derived from groundwater (the remaining 20% was derived from reclaimed wastewater with relatively low quality), and the diverted water has not been used for agriculture due to its relatively high price.

### How water diversion contributed to groundwater recovery

**Fig. 4:**
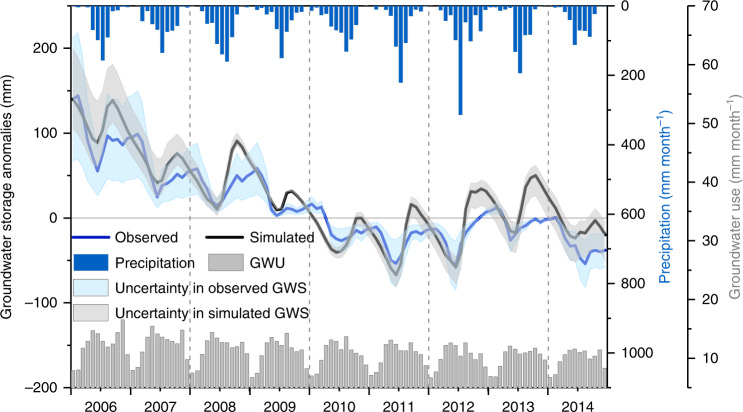
GWS anomalies, precipitation, and GW use for 2006‒2014. Uncertainty in simulated monthly GWS was estimated based on varying aquifer thicknesses from 100 to 250 m derived from aquifer properties. Uncertainty in observed GWS was approximated by the standard deviation of specific yield values (0.042) from 25 recorded wells across the Beijing Plain. The model calibration period spans from 2006 to 2010 and the validation period spans from 2010 to 2014. *R*^2^ between the simulated (trend: 13.1 mm year^−1^) and observed (trend: 14.4 mm year^−1^) GWS changes for the validation period is 0.85.

**Fig. 5:**
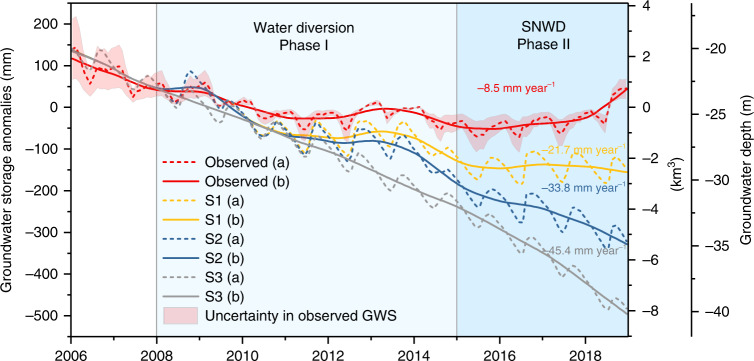
Impact of factors on GWS recovery in Beijing for 2006‒2018. GWS anomalies and decomposed time series using the STL (Seasonal and Trend decomposition using Loess) decomposition method from in situ measurements and GWS simulations from the CWatM-MODFLOW model during 2006‒2018 under three scenarios (S1‒S3). Shown also are linear GWD changing rates for each decomposed time series, with the significance of these rates listed at the 0.05 level (referring to the “Methods” section).

To explore the drivers of GWS changes behind the data collected and analyzed above, we set up a high-resolution hydrologic model (CWatM-MODFLOW) to quantify GWS changes in Beijing (the “Methods” section), which reproduces long-term trends, and interannual and seasonal variability in GWS well (Fig. 4). Differences between simulated and observed GWS changes are attributed to modeling deficiencies (e.g., uncertainties in aquifer thickness) and to uncertainties in observed GWS from spatial variability in specific yield. To quantify the relative contributions of water diversion, suppression of agricultural water use, and precipitation variability on GWS, we simulated three different scenarios (Fig. 5). Note that one layer of the confined aquifer was set in the model for simplifying the complex groundwater system, and we assumed that the estimated groundwater depletion under these scenarios was not limited by the actual groundwater storage. This quantification was conducted on GWS time series after removing the seasonal variability to minimize the impacts of seasonal variability of simulated and observed GWS on the trend analysis (see the “Methods” section for details).

Scenario I (S1): no water diversion during 2008–2014 and no water diversion for domestic and industrial use during 2015–2018 simulated by substituting diverted water by GW withdrawal. Without the diverted water, water use for the domestic and industrial sectors would have been derived from groundwater. S1 removes the benefits of increased water supply from the SNWD.

Scenario II (S2) builds on scenario 1 and assumes no water diversion and suppression of agricultural water use by simulating GW withdrawal for agricultural use during 2009–2018 remaining the same as that in 2008. S2 removes the benefits of increased water supply from the SNWD and the effects of the policy of limiting agricultural water use.

Scenario III (S3) builds on scenarios I and II and assumes no variability in precipitation by substituting actual precipitation during 2008–2018 by precipitation climatology during 2000–2018. S3 removes the benefits of the SNWD, agricultural water use policy, and increased groundwater recharge due to climate variability.

Results indicate that without water diversion GWS would decline at −21.7 mm year^−1^ (−0.36 km^3^ year^−1^, calculated by multiplying the GWS equivalent water thickness by the area of Beijing) during 2006–2018 under S1; without water diversion and suppression of agricultural water use, GWS would decline at −33.8 mm year^−1^ (−0.55 km^3^ year^−1^) under S2; and without water diversion, suppression of agricultural water use, and precipitation reduced to the precipitation climatology, GWS would decline by −45.4 mm year^−1^ (−0.75 km^3^ year^−1^) under S3, compared to the actual depletion rate of −8.5 mm year^−1^ (−0.14 km^3^ year^−1^) during 2006–2018 (Fig. 5). Specifically, during 2006–2018, water diverted to Beijing reduced GWD by ~3.7 km^3^ (calculated by the difference between the simulated GWS for S1 and observed GWS at the end of 2018), accounting for 40% of total GWS recovery. Suppression of agricultural water use reduced GWD by ~2.8 km^3^ (calculated by the difference between the simulated GWS for S2 and simulated GWS for S1 at the end of 2018), accounting for 30% of total GWS recovery. Increased precipitation reduced GWD by ~2.7 km^3^ (calculated by the difference between the simulated GWS for S3 and simulated GWS for S2), accounting for 30% of total GWS recovery. Overall, the central SNWD, along with the suppression of agricultural water use and precipitation variability, has reversed the GW level decline.

### How will Beijing’s groundwater storage change in the future

**Fig. 6:**
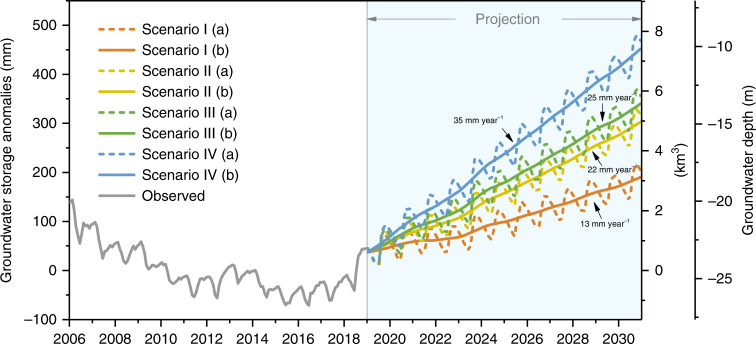
GW level projection under precipitation and GW use scenarios. The observed GWS anomalies are relative to the mean during 2006‒2018 and their decomposed time series (2019‒2030) under four scenarios. Scenario I: normal GW use (G1) + precipitation climatology (P1); Scenario II: low GW use (G2) + P1; Scenario III: G1 + a wet climate (P2); and Scenario IV: G2 + P2. Solid lines are observed or simulated GWS anomalies, and dashed lines are corresponding decomposed time series (see the “Methods” section).

Projections of GWS for Beijing were based on simulations of four scenarios considering precipitation and GW withdrawal during 2019−2030 (Supplementary Table 2). Variations in climate forcing were based on (I) precipitation climatology (P1: MAP during 2000−2018: 540 mm year^−1^) or (II) a wet climate (P2: MAP during 2008−2018: 580 mm year^−1^). Variations in human water use were based on annual GW pumping set equal to (I) GW withdrawal in 2018 (G1: 1.7 km^3^ year^−1^) or (II) 90% of it (G2: 1.5 km^3^ year^−1^). Results indicate that GWS in Beijing is likely to recover over the next decade under all these scenarios (Fig. 6), provided there will be at least 1 km^3^ year^−1^ of water diverted up to 2030. Based on the 13th Five-Year Development Plan for Beijing^43^ and the Overall City Planning for Beijing (2016–2035)^44^, the total number of residents will be strictly controlled to within 23 million by 2020 and maintained at this level over the long term (2020–2035). In fact, the number of residents in Beijing began to decline in 2017 (2017: 21.7 million; 2018: 21.5 million; 2019: 21.5 million) due to policies for relieving population pressure and related to the capital’s functions.

The most rapid recovery of GWS results from scenario IV, i.e., further reduction in GW withdrawal compared with that in 2018 under a wet climate, in which GWS is likely to recover to the 1990s GW depth of ~10 m (Fig. 1b) by 2030. The lowest recovery of GWS results from scenario I, i.e., stable GW withdrawal from 2018 under precipitation climatology, for which GWS would recover to the 2003 GW depth of ~18 m by ~2030. General circulation models show that precipitation in North China^45^ and Beijing is likely to increase during the next decade (Supplementary Fig. 4). Projected precipitation increases suggest a higher possibility of rapid GW recovery in these scenario simulations. Though GW is projected to recover over the long term, GW decline is likely to occur during climate extremes, such as droughts and heatwaves in Beijing and/or in the source region in the future. Continued GW recovery would also result in other environmental problems, such as salinization and waste of water.

As the largest water diversion project globally, the central SNWD exemplifies the combined use of SW and GW. Overall, the diversion has improved SW supply, resilience to climate extremes, the environment, and has contributed to recovery of GWS in Beijing. The planned diverted water volume (~9.5 km^3^ year^−1^) from the central route represents ~30% of mean annual streamflow into the Danjiangkou Reservoir in the source area. Long-term monitoring and research, as well as improved legislation, policies, and management practices are required to minimize negative environmental impacts in the source region^46,47^, particularly the resilience of water supply from the source region under future climate change and extremes, and seawater intrusion into estuaries of the Lower Yangtze River during low-flows due to water abstractions^48,49^.

Socio-environmental impacts of these large-scale water diversions also need to be considered. In the Danjiangkou Reservoir catchment, more than 300,000 people have been resettled for this project. Pesticide and fertilizer applications for agriculture have been limited and polluting industries are not allowed, restricting socioeconomic development in the source region. Navigation and environmental flows downstream of the Danjiangkou Reservoir were negatively impacted from the water diversions^50^. In addition, high prices of diverted water are an issue in water-receiving areas. To cover construction and maintenance costs of the central SNWD route, users have to pay higher prices for the water, increasing economic pressures on local governments, factories, residents, and farmers. The tradeoffs between increased water reliability within the context of climate extremes need to be considered relative to the socioeconomic impacts and costs of water diversions in the source region as construction of all of the SNWD routes are being finalized.

## Methods

### High-resolution Community Water Model coupled with MODFLOW

We set up a high-resolution Community Water Model (CWatM) of 30 arcsec spatial resolution (~1 km at the equator). CWatM was developed by the International Institute for Applied Systems Analysis (IIASA) Water Program to assess water demand and water availability influenced by climate and socioeconomic changes globally and regionally. CWatM is equipped with sophisticated modules (particularly the water demand module in this study) which are prerequisites to quantify the influence of human activities (e.g., water diversion and irrigation) on GW in Beijing. The high-resolution CWatM model with corresponding input data at 30 arcsec can better represent the spatial heterogeneity and describe the hydrologic processes at regional scales. Furthermore, the coupled CWatM model with MODFLOW (CWatM-MODFLOW)^51^, incorporating GW pumping and lateral flow, is more applicable to simulate GWS change in Beijing, because a portion of GW recharge in the piedmont plain is derived from mountain front recharge from surrounding Taihang and Yan mountains in the NCP^52^.

The coupled MODFLOW model has a relatively higher spatial resolution of 500 m to distinguish different areas of GW recharge and capillary rise (i.e., where GW levels in MODFLOW grid cells reach the lower soil layer of CWatM)^53^. In this study, we used one layer of the confined aquifer with an initial thickness of 200 m, which was estimated from contour maps of GW aquifer thickness in Beijing. Twenty-five wells from China Groundwater Level Yearbooks for Geo-environmental Monitoring were set as the pumping locations. Dynamic pumping rates were adopted, which were derived from monthly GW use data in Beijing. Before simulating the GWS, a steady-state model was run using annual average meteorological forcing data during 2000–2018 and the model was run 500 times. Then MODFLOW was triggered every 2 weeks to interact with CWatM bidirectionally: (1) GW recharge from CWatM to MODFLOW and (2) capillary rise and baseflow simulated by MODFLOW to the lower soil layer and the river network system in CWatM.

### Adjustment of simulated percolation

We simulated GWS in Beijing and examined GW recharge, which consists of two parts: (1) percolation from the soil layer to GW and (2) preferential flow (i.e., rapid GW recharge through holes and cracks in the soil). We found that the magnitude of percolation is too small relative to preferential flow, indicating that GW recharge is dominated by preferential flow in the original model. Thus, simulation of GW recharge was adjusted using an exponential equation (Eq. (1)) for calculating unsaturated hydraulic conductivity which has been examined in the NCP^54^. Other equations for calculating unsaturated hydraulic conductivity have been examined in previous research^55^.

1 $$K\left( \theta \right) = K_{\mathrm{s}} \, {\times} \exp \left( { - \beta \, {\times} \, \frac{{\theta _{\mathrm{s}} - \theta }}{{\theta _{\mathrm{s}}}}} \right),$$

where *K*_s_ is saturated hydraulic conductivity (m s^−1^); *θ*_s_ is volumetric moisture content at saturation (m^3^ m^−3^); *θ* is volumetric soil moisture content (m^3^ m^−3^); and *β* is a dimensionless constant representing soil heterogeneity and is further adjusted during calibration.

### Model calibration

We calibrated the model for monthly GWS changes for the 2006–2010 period using observed monthly GWS changes and the Non-dominated Sorting Genetic Algorithm II (NSGA-II) which is embedded in the distributed evolutionary algorithm in Python (DEAP)^56^. Multiobjective functions were adopted during calibration, including correlation coefficient and trends between the simulated and observed GWS time series. Observed GWS anomalies were derived from in situ measurements of GW depth and specific yield values for the GW aquifer. During calibration, parameters associated with snowmelt, evapotranspiration, infiltration, soil depth, interflow, preferential flow, soil heterogeneity, and the GW recession coefficient were adjusted automatically. After calibration, a Pareto front in terms of the two objective functions was derived. Then we set a simple metric *M* to select optimal parameters for GWS simulation (Eq. (2)). Given that our main concern was to have the accurate trend of modeled GWS, a higher weight of 0.7 was allocated to the simulated GWS trend.

2 $$M = 0.7 \times D + 0.3 \, {\times} \left( {1 - r} \right),$$

where *M* is the metric for selection of the optimal parameter set, and a smaller value of *M* indicates that the corresponding simulation has a better performance, dimensionless; *D* denotes the difference in the slopes of linear fit between the simulated and observed monthly GWS time series (L T^−1^) that is normalized using Eq. (3), dimensionless; and *r* is the correlation coefficient between the simulated and observed GWS time series, dimensionless.

Given that *D* and *r* are on different scales, to use the metric *M*, we normalized *D* and *r* between the simulated and observed GWS (Eq. (3)) so that they would range between 0 and 1.

3 $$x_{{\mathrm{{normalized}}}} = \frac{{x_i - x_{\min }}}{{x_{\max } - x_{\min }}},$$

where *x*_normalized_ denotes the normalized *D* (or *r*) value; *x*_*i*_ denotes the original value of *D* (or *r*); *X*_min_ denotes the minimum *D* (or *r*) among all the calibration results; and *X*_max_ denotes the maximum *D* (or *r*) among all the calibration results.

To reduce the influence of intra-annual variability and time delay of observed GWS peaks relative to simulated GWS peaks, we implemented a Seasonal and Trend decomposition using Loess (STL) to remove the seasonal signal of the GWS time series. After decomposition, the original time series was divided into three parts: trend, seasonal signal, and the remainder. Evaluation of the GW recovery and contributions of water diversion, suppression of agricultural water use, and climate variability was performed based on the decomposed GWS time series without the seasonal signal and remainder parts.

### Groundwater use and precipitation scenarios for 2019–2030

To predict changes in Beijing’s GWS during 2019–2030, both precipitation and groundwater use scenarios need to be reasonably formulated as two critical inputs to drive the model. As illustrated in the main text, reductions in GW withdrawal over the recent decade, particularly since the central SNWD route operated, were driven primarily by replacing GW withdrawals with diverted water for domestic and industrial use and reductions in agricultural water use. Based on the 13th Five-Year Development Plan (2016–2020) for water resources in Beijing^43^ and assuming that the resulting GW withdrawal in 2018 or a stricter reduction (the GW withdrawal in 2018 multiplied by a factor of 0.9) will persist in the following decade 2021–2030 (i.e., the 14th (2021–2025) and 15th (2026–2030) Five-Year Development Plans), we formulated four scenarios considering both future GW withdrawal and precipitation (Supplementary Table 2) for predicting GWS changes in Beijing by 2030.

Two scenarios for GW use were formulated: (G1) GW pumping during 2019–2030 was the same as that in 2018 (i.e., GW pumping of 101 mm year^−1^ or 1.7 km^3^ year^−1^) and (G2) GW pumping during 2019–2030 was further reduced relative to that in 2018 (i.e., 91 mm year^−1^ or 1.5 km^3^ year^−1^, a multiplier of 0.9). Two scenarios for precipitation were formulated: (P1) monthly precipitation during 2019–2030 was set to the climatology of in situ monthly precipitation during 2000–2018, resulting in annual precipitation of 540 mm year^−1^, and (P2) monthly precipitation during 2019–2030 was set to a wet climate of in situ monthly precipitation during 2008–2018, resulting in annual precipitation of 580 mm year^−1^. Scenario I was the combination of G1 and P1; Scenario II was the combination of G2 and P1; Scenario III was the combination of G1 and P2; and Scenario IV was the combination of G2 and P2. Meteorological data used to force CWatM-MODFLOW were derived from regional climate model (RCM) DMI-HIRHAM5 under RCP4.5 (representative concentration pathway, RCP) within the Coordinated Regional Climate Downscaling Experiment (CORDEX) framework. Precipitation data from RCM containing interannual and intra-annual variability were adopted, with monthly RCM precipitation corrected to either the climatology of in situ monthly precipitation during 2000–2018 (i.e., 540 mm year^−1^, P1) or to a wet climate during 2008–2018 (i.e., 580 mm year^−1^, P2) using multiplicative factors. The RCM data were interpolated to a 30 arcsec spatial resolution. To incorporate interannual variability in RCM precipitation in the projections of GWS changes for future scenarios, we also conducted another set of projections (Supplementary Note 3 and Supplementary Fig. 5 for details). The finding of overall increasing trends in GWS in Beijing in the coming decade did not change.

Note that there is a strong possibility that the government will impose stricter constraints on GW withdrawal during the 2021–2030 decade compared to that presented in the 13th Five-Year Development Plan. There is a possibility to further increase water diversion from the central SNWD route by 0.1–0.2 km^3^ year^−1^ during the next decade. Furthermore, the eastern SNWD route plans to provide an additional 0.3–0.5 km^3^ year^−1^ through a canal from Tianjin to Beijing from 2030. We assumed that the increased amount of the domestic water use will be compensated for by increased water diversion. In addition to these favorable conditions for increasing diverted water, GWS in Beijing is likely to recover faster during the next decade due to the stricter policies on reducing demand for new water resources and increasing the security of water supply, including further reduction in agricultural water use, replacement of GW withdrawal with diverted water for domestic and industrial use, and GW replenishment by diverted water that is not currently considered in the model. But GW supply can still be very critical for easing water supply shortages during droughts due to its ubiquity, flexibility, and lower costs, as exemplified in the 2014 summer drought. More sustainable forms of water use and management can therefore be realized by better combining use of GW and SW, including diverted water in the near future.

## Supplementary information

Supplementary Information

Peer Review File

## Data Availability

Data used in this study include ground-based monitoring, statistical data, and reanalysis data. Monthly GW depth measurements for Beijing during 2005–2019 were derived from Beijing Groundwater Dynamics Bulletins provided by the Beijing Water Authority^26^. A specific yield of 0.081 was derived from Beijing Water Resources Bulletins to estimate GWS changes using GW depth measurements made at 110 wells in the Beijing Plain (Supplementary Fig. 1). Monthly water use data for 2006–2018 in terms of various water sources (i.e., SW, GW (self-contained wells and water-source wells), reclaimed water, and diverted water) and sectors (i.e., agricultural, domestic, industrial, and environmental use) were provided by the Beijing Water Authority. Annual water use data in terms of various water sources and sectors were derived from Beijing Water Resource Statistics Year Books^57^. GW depth, water supply, and water use data can be downloaded from https://figshare.com/articles/Beijing_Data_xlsx/5952394/1 or are available from the first author upon request. SW (mainly reservoirs) storage data (Fig. 1c) were derived from Annual Beijing Water Monitoring Bulletins^58^ or can be accessed from the website of the Beijing Water Authority (http://nsbd.swj.beijing.gov.cn/dzxsksq.html). Basic inputs of CWatM include maps or parameters of land cover, soil, topography, lakes, reservoirs, GW, water demand, and routing, which were derived from previous studies and data sets^59–65^. Meteorological forcing data include precipitation, humidity, longwave/shortwave downward radiation fluxes, maximum/minimum/average 2-m temperature, 10-m wind speed, and surface pressure, for the default calculation method for evapotranspiration (i.e., the Penman-Monteith method)^66–73^. Links to access all the input data sources of the model were put into an Excel file generated by Google Sheets (https://docs.google.com/spreadsheets/d/1D6iwrIN-Z6RAt6h5Hs_veH5kNX4DJZDVxmQ2VLdQHh0/edit?usp=sharing). All input data under different spatial resolutions were interpolated to a 30 arcsec spatial resolution using the bilinear interpolation method.

## Code availability

The CWatM model is a fully open-source model available at https://cwatm.iiasa.ac.at/. Codes used to produce this work are available upon request.
