# Supplementary material for: South-to-North Water Diversion stabilizing Beijing’s groundwater levels
Source: Nat Commun. 2020 Jul 21;11:3665. doi: 10.1038/s41467-020-17428-6 (PMC7374106; doi:10.1038/s41467-020-17428-6)
Supplement: Supplementary file 1 — Supplementary Information [file 41467_2020_17428_MOESM1_ESM.pdf]

Supplementary Information for: “South-to-North Water Diversion stabilizing Beijing's groundwater levels” by Long et al.

1. Supplementary Notes

Supplementary Note 1. Details on the South-to-North Water Diversion Project

The South-to-North Water Diversion (SNWD) Project was launched in 2002 by the Chinese government to transfer water from the Yangtze River in the south of China to the drier north through three canal and pipeline systems. The eastern route, designed to transfer 14.8 km^3 of water annually, started to transfer water from Yangzhou City (i.e., downstream of the Yangtze River) through Jiangsu, Anhui, Shandong, and Hebei Provinces and Tianjin Municipality since 2013. The central route, designed to transport 9.5 km^3 of water annually from the Danjiangkou Reservoir in the middle reaches of the Hanjiang River (i.e., the largest tributary of the Yangtze River) through Henan and Hebei Provinces, and Beijing and Tianjin Municipalities began to operate since Dec 2014. The western route, planned to divert 20 km^3 from the upper reaches of the Yangtze River (i.e., Tongtian, Yalong, and Dadu Rivers) to the Yellow River in northwest China is still being planned and has yet started to transport water.

Supplementary Note 2. Details on water diversion to Beijing

There are two phases of water diversion to Beijing discussed in this study. During phase I (2008–2014), major reservoirs (i.e., Gangnan, Huangbizhuang, and Wangkuai) in Hebei Province supplied 1.6 km^3 of water to Beijing, which was not included in the initial plan of the central SNWD route. During phase II (Dec 2014–Dec 2019) when the principal part of the central SNWD route operated, a total of ~ 26 km^3 of water has been transported to North China, of which 5.2 km^3 (~ 1 km^3 yr^{-1}) reached Beijing (the amount of 1 km^3 yr^{-1} accounting for 26% of annual total water use of 3.8 km^3 in Beijing). This has profoundly altered the water supply structure and indirectly impacted groundwater storage (GWS) there.

Supplementary Note 3. Impacts of interannual variability in precipitation on GWS projection

We also projected GWS changes by incorporating interannual variability in precipitation. Two new precipitation scenarios during 2019–2030 were generated based on the original RCM precipitation: (P1N) original RCM annual precipitation during 2019–2030 (mean annual precipitation of 750 mm yr^{-1}) multiplied by a factor of 0.72 (540/750), to represent the climatology (i.e., mean annual precipitation during 2000–2018, 540 mm yr^{-1}), and (P2N) original RCM annual precipitation during 2019–2030 multiplied by a factor of 0.77 (580/750), to represent a wet climate (i.e., mean annual precipitation during 2008–2018, 580 mm yr^{-1}). Groundwater use scenarios are the same as the initial projection.

New projections of GWS during 2019–2030 clearly show the impacts of interannual variability in precipitation on GWS change and higher rates of the recovery than the

initial projections (Supplementary Figure 5). The lowest recovery rate was estimated to be 28 mm yr^{-1} (groundwater depth of ~ 13 m in 2030) under scenario I, and the highest recovery rate was found to be 50 mm yr^{-1} (groundwater depth of ~ 5 m in 2030) under scenario IV. However, these trends may have been overestimated, because increased groundwater withdrawal during drought was not incorporated into these scenarios. For instance, annual precipitation in 2026 was estimated to be 282 mm yr^{-1} (P1N) after bias correction, which is much less than the historical precipitation level and would likely result in substantial groundwater pumping to alleviate the drought. However, the finding of overall increasing trends in GWS in Beijing in the coming decade did not change.

2. Supplementary Tables

Supplementary Table 1. Shallow GWS trends of major cities in the North China Plain during 2003–2010. These statistical data were derived of *Groundwater Bulletin of China Northern Plains* and *Groundwater Bulletin of the Hebei Plain*.

City	GWS trend/(cm yr^{-1})
Beijing	-7.54
Tianjin	0.07
Handan	-4.32
Xingtai	-2.78
Shijiazhuang	-8.89
Baoding	-4.24
Langfang	-1.19
Tangshan	-0.21
Qinhuangdao	1.11

Supplementary Table 2. Groundwater withdrawal and precipitation scenarios for Beijing during 2019–2030. Four scenarios were formulated considering both groundwater withdrawal reductions and precipitation changes, i.e., (1) G1+P1; (2) G2+P1; (3) G1+P2; and (4) G2 +P2.

Scenario		Illustration	**Abbrev.**
Groundwater pumping and water use	Normal condition	Groundwater pumping and water use during 2019–2030 are the same as in 2018 (i.e., groundwater pumping of 101 mm (1.7 km^3) yr^{-1})	G1
	A lower condition	Groundwater pumping during 2019–2030 is further decreased relative to that in 2018 (i.e., 91 mm (1.5 km^3) yr^{-1}, we used a multiplier of 0.9)	G2
Precipitation	Climatology	Monthly precipitation during 2019–2030 is set to the climatology of in situ monthly precipitation during 2000–2018, resulting in annual precipitation of 540 mm yr^{-1}	P1

	A wetter climate	Monthly precipitation during 2019–2030 is set to the climatology of in situ monthly precipitation during 2008–2018, resulting in annual precipitation of 580 mm yr^{-1}	P2

3. Supplementary Figures

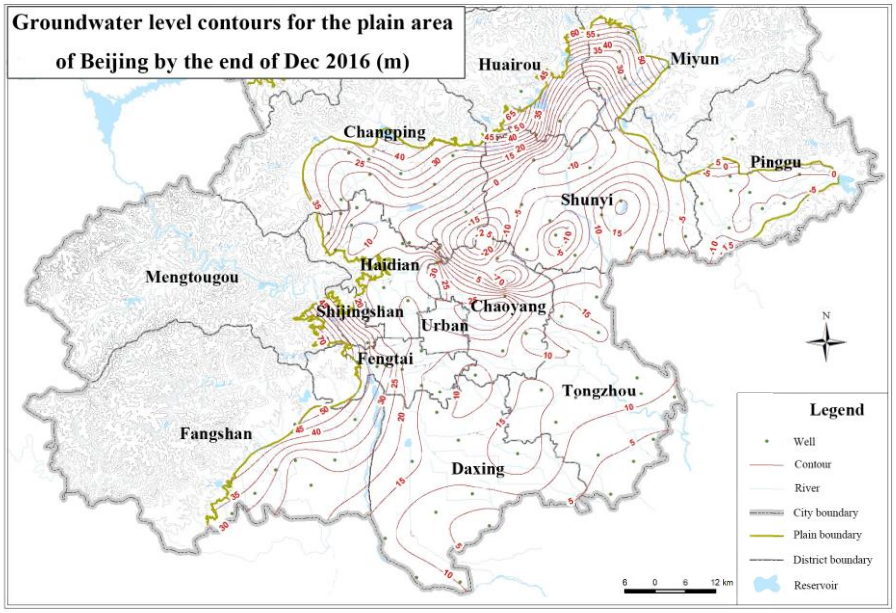

Supplementary Figure 1| Locations of 110 wells and groundwater level contours for Beijing's Plain by the end of Dec 2016 (m), adapted from Beijing Monthly Groundwater Bulletin provided by the Beijing Water Authority.

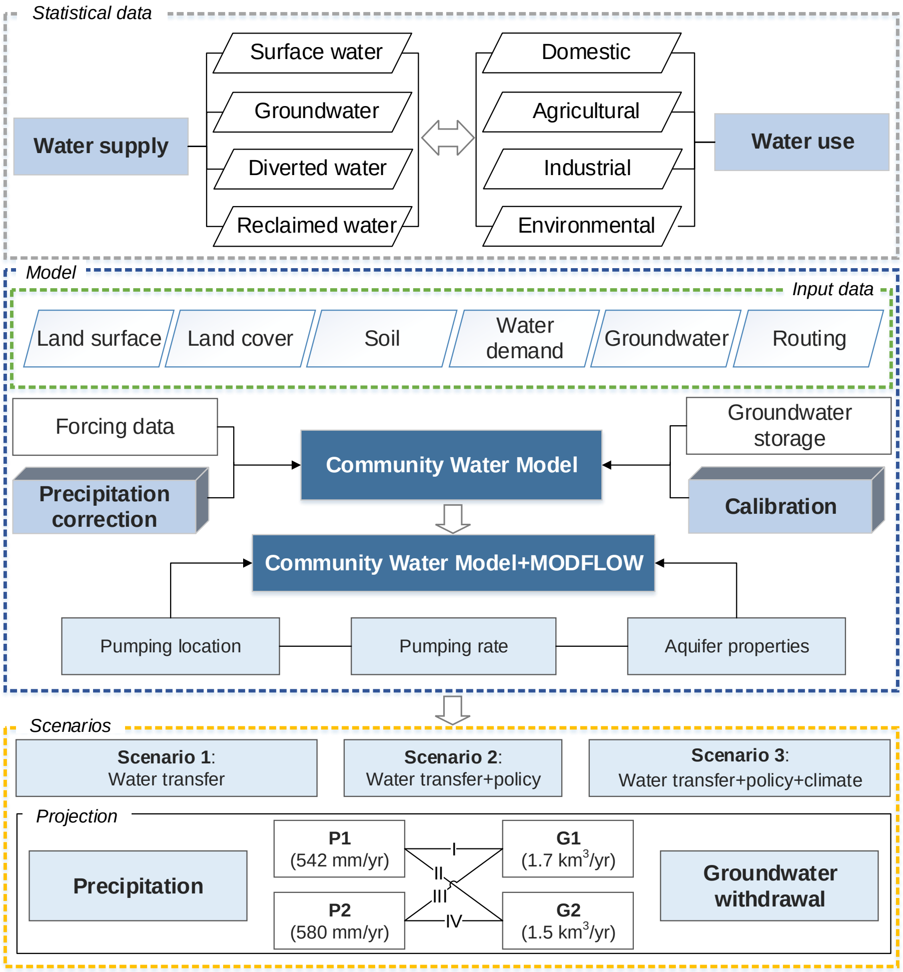

Supplementary Figure 2| Flowchart of the methodology of this study, including data (i.e., statistic data and model inputs), model (the Community Water Model and MODFLOW), and scenarios (for quantifying contributions of water diversion, policies, and climate variability to groundwater recovery) we used for analyzing and predicting groundwater storage changes in Beijing.

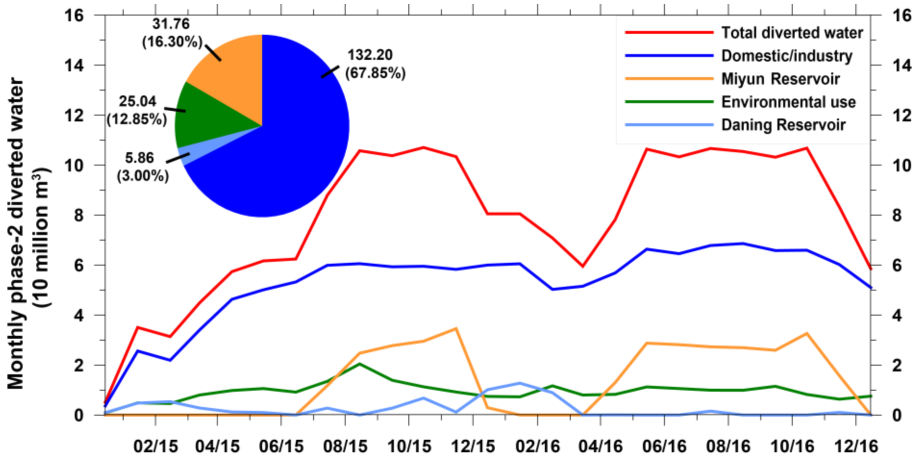

Supplementary Figure 3| Monthly diverted water, showing use and percentage (pie chart; units 10 million

m^3) for the period Dec 2014–Dec 2016. Water use includes the proportions: (1) transferred to waterworks for domestic and industrial use, (2) stored in the Miyun Reservoir, (3) for environmental use, and (4) stored in the Daning Reservoir. The fractions of different water use categories supplied by the central SNWD route for the five-year period from Jan 2015 to Dec 2019 are similar to the two-year period from Dec 2014 to Dec 2016 when monthly diverted water data are available for this analysis. Annual data and the fractions for the three-year period from Jan 2017 to Dec 2019 are available for this analysis.

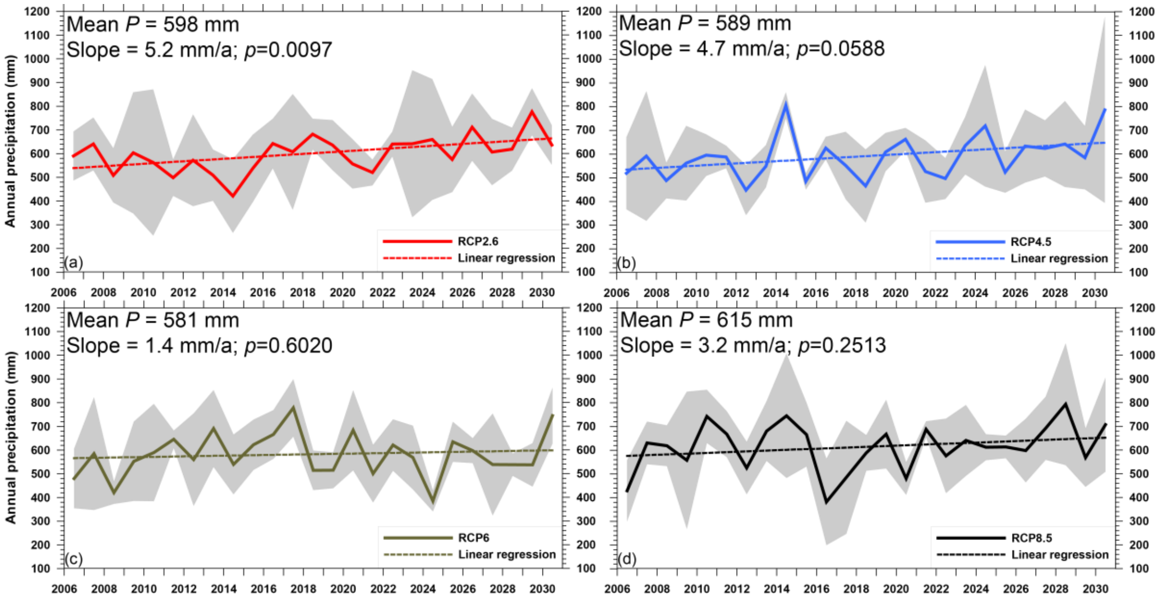

Supplementary Figure 4| Mean projected annual precipitation from three general circulation models (GCMs) outputs (GFDL-ESM2M, HadGEM2-ES, and IPSL-CM5A-LR) under four representative concentration pathways (RCPs: (a) RCP2.6, (b) RCP4.5, (c) RCP6, and (d) RCP8.5) from the Inter-Sectoral Impact Model Intercomparison Project (ISI-MIP) for Beijing during 2006–2030. Backgrounds show uncertainties approximated by one standard deviation from the three GCMs for each RCP. This figure also shows mean annual precipitation (P) for the study period 2006–2030, and slope and p-values of linear regression for annual precipitation time series for each RCP.

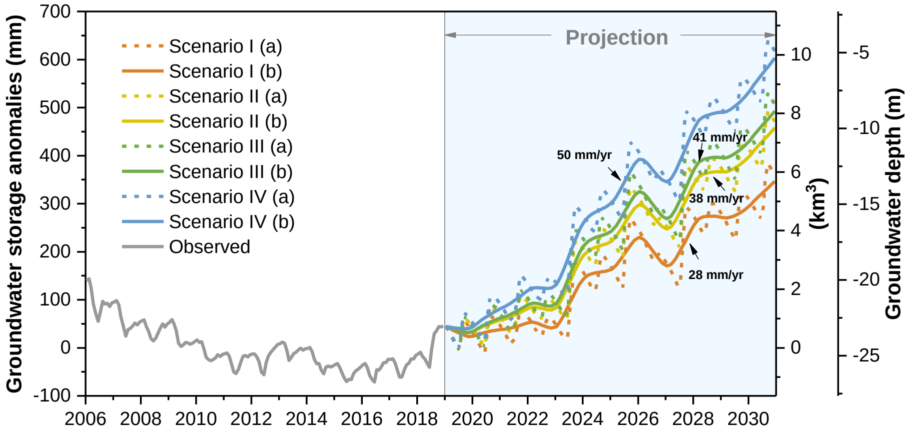

Supplementary Figure 5| Projections of GWS change under GW use (normal and low) and precipitation

(climatology (P1N) and a wetter climate (P2N)) scenarios in Beijing during 2019–2030. Same as Fig.6 in the main text but with new precipitation scenarios (i.e., P1N and P2N, Supplementary Note 3).
