# Supplementary material for: South-to-North Water Diversion stabilizing Beijing’s groundwater levels
Source: Nat Commun. 2020 Jul 21;11:3665. doi: 10.1038/s41467-020-17428-6 (PMC7374106; doi:10.1038/s41467-020-17428-6)
Supplement: Supplementary file 2 — Peer Review File [file 41467_2020_17428_MOESM2_ESM.docx]

Peer Review Information

**Journal:** Nature Communications

**Manuscript title:** South-to-North Water Diversion Stabilizing Beijing's Groundwater Levels

**Corresponding author name(s):** Dr Di Long

**Editorial notes:**

**Transferred manuscripts**

This manuscript has been previously reviewed at another journal that is not operating a transparent peer review scheme. This document only contains reviewer comments and rebuttal letters for versions considered at *Nature Communications*.

**Reviewer comments & decisions:**

**Reviewer comments, first version:**

Reviewer #1 (Remarks to the Author):

The authors provide a thoughtful and interesting evaluation of the impact of a variety of policy and climate drivers on local water budgets, with a focus on groundwater, in the North China Plain (NCP). They explore the impacts of the South-to-North Water Diversion project as well as other policy actions (i.e. strict limitations on groundwater abstraction for agriculture) and precipitation variability to explain trends in groundwater recharge through the NCP. The authors’ analysis offers valuable insight to the relative influence of these drivers on groundwater levels over time. I found it very interesting that the influence of the SNWD project, the policy change to limit agriculture groundwater use, and increased precipitation each contributed about a third to groundwater recharge in the North China Plain. The paper speaks to the benefit of a multi-faceted policy toolkit to sustainably manage groundwater reousrces. Overall, the manuscript is well-written, the data analysis is robust, and the presentation in the figures is easy to interpret.

I have a few suggestions and points of clarification before I feel comfortable suggesting the manuscript be published.

First and foremost, from how I am interpreting it, the baseline scenario (S1) that is modeled in the paper removes the benefits of increased water supply from the SNWD project from 2015-2018 by increasing the substituting the equivalent volume as groundwater withdrawal allocates this water to domestic and industrial supply. Some additional information about this scenario would be useful. Is it valid to assume that all water from SNWD would be allocated to meet domestic and industrial water demand? I’m just curious about how the SNWD water is allocated and if that is a valid assumption to make. Some clarification about the baseline scenario and discussion of how the SNWD water is allocated would be useful.

Figure 2 indicates that water demand is increasing. If urban population in Beijing is actually declining, does the increase in domestic water use indicate an intensification of domestic water use? What is driving that increase and how was that accounted for in the model to predict future groundwater changes in the NCP?

Figure 4 highlights differences in the observed and simulated GW storage anomalies. The modeled results to have a greater magnitude of interannual variability as compared to the observed groundwater storage changes. The model appears to consistently overestimate groundwater storage anomalies. Can the authors provide some clarification or explanation of the discrepancies between the modeled and observed groundwater storage patterns? Additionally, the authors should provide a clarification for how this may affect their results.

Some minor comments and suggested edits:

Figure 3(b) shows a dramatic drop in domestic water use and increase in environmental water use in 2015. What is driving that trend? It seems to be a very notable anomaly that is not addressed or discussed in the manuscript.

Can the authors clarify somewhere in the manuscript the difference between “self-contained wells” and “water-source wells”?

Thank you for your time and consideration of my comments.

Sincerely,
Katalyn Voss


Reviewer #2 (Remarks to the Author):

General comments

Groundwater depletion in the North China Plain has been long concerned. The South-North Water Transfer (SNWT) Project has been implemented as one of the countermeasures, and the effect is being emerged. This study quantitatively analyzed the contribution of the project and other policies to the recovery of groundwater level near Beijing over past 20 years by data analysis and elaborative model simulation.
The manuscript is well structured and successful to demonstrate the relationship between the policies and groundwater level comprehensively. The major concern I observed as a scientific report is that the major findings and conclusions (i.e. the groundwater recovery is attributed to SNWT, agricultural water saving, and increasing trend of precipitation) can be solely derived from the data provided by the Beijing Water Authority and other local governmental agencies. I was not able to perceive that much information was additionally earned from the model simulation which is the key original contribution of the authors.

Specific comments

Line 125 “… were examined”: I suppose the authors conducted this analysis using the monitored data of Beijing Water Authority (BWA). Hence all the numbers shown in this section can be taken as the known facts assuming all the data were properly observed and reported. This point should be clearly mentioned here.

Line 200 “Results indicate that …”: I found the findings from the model simulation are largely overlap with the ‘known facts’ shown in the previous subsection. I am wondering what the new insights are the authors’ model has added.

Line 208 “Overall, the central SNWD route, along with suppression of agricultural water use and precipitation variability has reversed the GW decline”: Again, these key features are already clearly seen the data by BWA (Figures 1 and 2). I could not figure out what the authors originally explored through their modeling.

Line 357 “Two scenarios for precipitation were formulated … precipitation during 2019-2030 was set to the climatology of precipitation during 2000-2018”: How the sub-annual and inter-annual variations of precipitation were generated? I fully understand near-term climate projection is hard to be carried out, but these would certainly influence the projection of groundwater storage. For insightful discussion, the scenario design is better to be revisited, or at least further elaborated.

Technical comments

Fig 1 (b,c): Annotations and legends are too small.

Line 98 “Beijing has less than 100m3 per capita water resources”: I guess the number totally depends on the definition of areal extent of city. What is the definition of area for this case (e.g. administrative area or catchment area)?

Line 103 “derived from precipitation”. This phrase is unclear and confusing. Both groundwater and surface water are derived from precipitation in a sense.

**Author rebuttal, first version:**

Reviewer #1 (Remarks to the Author):

Comment R1C1: The authors provide a thoughtful and interesting evaluation of the impact of a variety of policy and climate drivers on local water budgets, with a focus on groundwater, in the North China Plain (NCP). They explore the impacts of the South-to-North Water Diversion project as well as other policy actions (i.e. strict limitations on groundwater abstraction for agriculture) and precipitation variability to explain trends in groundwater recharge through the NCP. The authors’ analysis offers valuable insight to the relative influence of these drivers on groundwater levels over time. I found it very interesting that the influence of the SNWD project, the policy change to limit agriculture groundwater use, and increased precipitation each contributed about a third to groundwater recharge in the North China Plain. The paper speaks to the benefit of a multi-faceted policy toolkit to sustainably manage groundwater resources. Overall, the manuscript is well-written, the data analysis is robust, and the presentation in the figures is easy to interpret.

Response to R1C1: We highly appreciate Reviewer #1 for providing the very encouraging evaluation and precise summary of this study.

I have a few suggestions and points of clarification before I feel comfortable suggesting the manuscript be published.

Comment R1C2: First and foremost, from how I am interpreting it, the baseline scenario (S1) that is modeled in the paper removes the benefits of increased water supply from the SNWD project from 2015‒2018 by increasing the substituting the equivalent volume as groundwater withdrawal allocates this water to domestic and industrial supply. Some additional information about this scenario would be useful. Is it valid to assume that all water from SNWD would be allocated to meet domestic and industrial water demand? I’m just curious about how the SNWD water is allocated and if that is a valid assumption to make. Some clarification about the baseline scenario and discussion of how the SNWD water is allocated would be useful.

Response to R1C2: Thank you for the comments. Your interpretation on scenario 1 (S1) is correct. The assumption that the major portion of the diverted water has been allocated to domestic and industrial water demand is based on the reported information and observed water use data. The diverted water from the central SNWD route (5.2 km^3^ during Dec 2014‒ Dec 2019) was allocated mainly for (1) domestic and industrial use after treatment at waterworks (~ 3.5 km^3^, 67%), and partially for (2) reservoirs (e.g., Miyun and Daning Reservoirs) (~ 0.7 km^3^, 14%) as a strategic water reserve; and for (3) environmental use (e.g., water replenishment for rivers and lakes) (~ 1 km^3^, 19%) (Please find Fig. S3 in the Supporting Information (SI) shown below). We did not assume that ALL diverted water would be substituted by groundwater withdrawal, but only the portion of the diverted water for domestic and industrial use (i.e., ~ 67% of the diverted water).

There are four major water use sectors examined in this study, including agricultural, domestic (i.e., households, the service sector, and construction), industrial, and environmental sectors. The waterworks mainly supply water for domestic and industrial use. Without the diverted water, this portion of water would have to be derived from groundwater based on historical data. More than 80% of agricultural water use is derived from groundwater with low cost (the remaining 20% is derived from reclaimed water of relatively low quality), and the diverted water has not been used for agriculture due to its relatively high price (see the last paragraph of this manuscript that discusses the issues of water diversion). Therefore, the assumption of S1 that the portion of the diverted water for domestic and industrial use would be substituted by groundwater is valid. We have supplemented the description of S1 in Lines 211−212 and the discussion on water allocation of the SNWD in Lines 192−194.

**Fig. S3| Monthly diverted water, showing use and percentage (pie chart; units 10 million m^3^) for the period Dec 2014‒Dec 2016.** Water use includes the proportions: (1) transferred to waterworks for domestic and industrial use, (2) stored in the Miyun Reservoir, (3) for environmental use, and (4) stored in the Daning Reservoir. The fractions of different water use categories supplied by the central SNWD route during Dec 2014‒Dec 2019 are similar to the period Dec 2014‒Dec 2016 when monthly diverted water data were available for this analysis. Annual data and the fractions for the period Jan 2017‒Dec 2019 were also available for this analysis.

Comment R1C3: Figure 2 indicates that water demand is increasing. If urban population in Beijing is actually declining, does the increase in domestic water use indicate an intensification of domestic water use? What is driving that increase and how was that accounted for in the model to predict future groundwater changes in the NCP?

Response to R1C3: Yes, you are correct; the increase in water use reflects an intensification of domestic water use. The urban population in Beijing peaked in 2017 and then maintained stable (2017: 21.7 million; 2018: 21.5 million; 2019: 21.5 million) as what we show in the manuscript (see Lines 262−263). However, domestic water use, including water use for households, the service sector, and construction all increased to a certain degree (domestic water use: **2016: 1.76 km^3^; 2017: 1.84 km^3^; 2018: 1.90 km^3^**). The slight increase in domestic water use was driven primarily by increased water use by households and the service sector as the social economy improved (e.g., increased incomes and residential areas, and elevating living standards). However, the rate of increase in domestic water use remained fairly stable (0.08 km^3^ in 2017 to 0.06 km^3^ 2018), indicating that domestic water use should not grow substantially if the current policies restricting the number of residents in Beijing and transferring the functions of the capital to other regions continue to be implemented (see Lines 160−166).

Regarding the projection of groundwater changes, we assumed that the increased amount of domestic water use will be compensated for by the increasing capability of water diversion by the SNWD project without largely impacting groundwater resources (Lines 411‒417). This is considered a reasonable assumption because the SNWD has been mainly supplying water for domestic use and the capability of water diversion continues to increase (diverting ~ 1 km^3^/yr currently to ~ 1.2 km^3^/yr in the later phase of the central SNWD route by 2030). In this study, the projection of groundwater storage changes mainly reflects the effect of groundwater substitution by the diverted water under future precipitation scenarios. We also indicate that groundwater could be a critical source for buffering the impact of extreme weather/climate events when there is insufficient surface water to divert from the source region (see Lines 279−281 and 417−419).

**Fig. 2| Annual and monthly water use in terms of (a, b) water source and (c, d) sector in Beijing for 2003–2018. a**, Annual water use (bars) in terms of different water sources (km^3^) and annual fractions of total water use (lines), including SW, GW, diverted water, and reclaimed water. **b**, Monthly water use in terms of different water sources (volume, million m^3^; equivalent water thickness, mm). **c**, Annual water use (bars) for different water use sectors (km^3^) and their annual fractions of total water use (lines), including domestic, agricultural, industrial, and environmental water use. **d**, Monthly water use of different water use sectors (volume, million m^3^; equivalent water thickness, mm)). 'Mon' refers to monthly in line graphs in Figs.2b and 2d.

Comment R1C4: Figure 4 highlights differences in the observed and simulated GW storage anomalies. The modeled results to have a greater magnitude of interannual variability as compared to the observed groundwater storage changes. The model appears to consistently overestimate groundwater storage anomalies. Can the authors provide some clarification or explanation of the discrepancies between the modeled and observed groundwater storage patterns? Additionally, the authors should provide a clarification for how this may affect their results.

Response to R1C4: The higher magnitude of modeled interannual groundwater storage variability relative to observed variability primarily reflects modeling deficiencies and uncertainties in observed GWS changes. Figure 4 in the original manuscript also illustrates the range of uncertainties in the modeled and observed groundwater storage, which were derived from uncertainties in aquifer thickness and specific yield. Aquifer thickness can influence the variability of the modeled groundwater storage, and specific yield can directly impact the calculation of observed groundwater storage change (groundwater storage = groundwater level multiplied by specific yield).

Within the range of uncertainty, interannual variability in modeled (gray shaded areas) and observed (blue shaded areas) groundwater storage anomalies is similar. We further discuss the difference of interannual variability between the modeled and observed groundwater storage in Lines 202−204. Given that our main concern was to have the accurate trend of modeled groundwater storage anomalies to quantify effects of different factors on groundwater storage change and to make reliable predictions in the future, the current simulated results were adopted with the lowest differences in the trends between the simulated and observed groundwater storage changes.

However, we acknowledge the difference between the observed and simulated groundwater storage anomalies, which has now been discussed as is illustrated above.

Some minor comments and suggested edits:

Comment R1C5: Figure 3(b) shows a dramatic drop in domestic water use and increase in environmental water use in 2015. What is driving that trend? It seems to be a very notable anomaly that is not addressed or discussed in the manuscript.

Response to R1C5: Thank you for identifying an error in our data. The anomalous changes in domestic and environmental water uses reflect an error made during data compilation. We apologize for this error. The file format that contains water use data in 2015 differs from that in other years. A column on water use from the service sector that should have been put into the domestic water use category was wrongly combined with environmental water use. Therefore, the sum of domestic and environmental uses remains unchanged, and this mistake does not impact the water use data for other years and from other sectors. We corrected it and revised this figure accordingly. Please see Figure 3(b) in the revised manuscript.

Comment R1C6: Can the authors clarify somewhere in the manuscript the difference between “self-contained wells” and “water-source wells”?

Response to R1C6: “Self-contained wells” refer to wells owned by some factories, mines, colleges, and farmers for their own use whereas “water-source wells” refer to wells owned by waterworks that supply water through the public pipe network system. We have further defined these terms in Lines 173−174 and 178−179.

Reviewer #2 (Remarks to the Author):

General comments

Comment R2C1: Groundwater depletion in the North China Plain has been long concerned. The South-North Water Transfer (SNWT) Project has been implemented as one of the countermeasures, and the effect is being emerged. This study quantitatively analyzed the contribution of the project and other policies to the recovery of groundwater level near Beijing over past 20 years by data analysis and elaborative model simulation.

Response to R2C1: We highly appreciate Reviewer #2 for providing the precise summary and comments of our manuscript.

Comment R2C2: The manuscript is well structured and successful to demonstrate the relationship between the policies and groundwater level comprehensively. The major concern I observed as a scientific report is that the major findings and conclusions (i.e. the groundwater recovery is attributed to SNWT, agricultural water saving, and increasing trend of precipitation) can be solely derived from the data provided by the Beijing Water Authority and other local governmental agencies. I was not able to perceive that much information was additionally earned from the model simulation which is the key original contribution of the authors.

Response to R2C2: It is true that prior to conducting the modeling, we had to perform comprehensive analyses of data from various authorities and agencies to identify potential drivers of groundwater storage changes in this region. This is a pre-requisite for appropriately designing the modeling scenarios such that impacts of different factors (i.e., SNWD, polices on suppressing agricultural water use, and climate variability) on groundwater storage recovery could be reasonably quantified.

Apparently, the knowledge on how different factors contribute to groundwater storage recovery is difficult to determine directly from the data alone. For instance, we have hydrologic flux data on precipitation, water use, and diverted water, and hydrological state data on groundwater levels. Though we can readily distinguish that precipitation in a year is above/below average, how much the variations in precipitation contribute to groundwater recovery and what the mechanism is cannot be fully understood by only looking at these data. A solid modeling framework is valuable in integrating all of these data to isolate the contributions of each driver to groundwater recovery. Furthermore, without the hydrologic model, we would not be able to predict future groundwater storage variations, as is reflected in this manuscript.

With a physical and mathematical description of groundwater recharge/inflows (i.e., processes of infiltration, percolation, and preferential flow) and discharge/outflows (i.e., groundwater pumping, baseflow, and capillary rise) in the model, we can clarify how and when precipitation reaches the aquifer, and subsequently conduct analyses to quantify the contributions of different factors to groundwater recovery. These quantitative data are essential and valuable for formulating plans of future water diversion and polices on water supply, which plays a critical role in the sustainability of the water resources system in Beijing.

Note that using statistical analysis (e.g., multiple linear regression) may also provide similar results, which is what we did for past versions of this manuscript that were reviewed multiple times in Nature journals, but as indicated by previous reviewers, the results were not considered defensible, underscoring the need for a solid modeling framework, particularly with water demand and groundwater pumping modules to separate different effects on groundwater recovery that were reflected in the in situ data.

We have spent more than two years to construct and set up a hydrologic model with local data and necessary calibration for hydrologic states (e.g., groundwater storage) and hydrologic fluxes (e.g., discharge) to improve the accuracy. The model simulation has been supported by the reported statistics obtained and processed as necessary. The reported statistics have been key information on designing our scenario assessments using the high-resolution hydrologic model for Beijing. Therefore, it is necessary to conduct the analysis with the modeling effort.

We have emphasized the role of the model and novelty in the revised manuscript in Lines 86−94.

Specific comments

Comment R2C3: Line 125 “… were examined”: I suppose the authors conducted this analysis using the monitored data of Beijing Water Authority (BWA). Hence all the numbers shown in this section can be taken as the known facts assuming all the data were properly observed and reported. This point should be clearly mentioned here.

Response to R2C3: The data are internally consistent and the gradual trends seem realistic. We have emphasized the source of data and the purpose of this examination in Lines 125−130.

Comment R2C4: Line 200 “Results indicate that …”: I found the findings from the model simulation are largely overlap with the ‘known facts’ shown in the previous subsection. I am wondering what the new insights are the authors’ model has added.

Response to R2C4: Our main scientific contribution stands for highlighting the mechanisms of complex groundwater storage changes in Beijing where various factors contribute to influencing the water resources. Not to mention, the SNWD is an additional major factor influencing the groundwater storage change. Our calibrated model simulation can show seamlessly the trend and underlying mechanisms of human activities (e.g., water use, irrigation, and pumping) and climate (e.g., precipitation, evaporation, and recharge) toward the near future. The national statistics have been useful to verify the results and fine-tune the model but does not provide future trajectories, which is necessary for a long-term water resources management to prepare for necessary adaptation measures. Our study tried to make a first step to construct such detailed set up that can be improved further to include mitigation and adaptation measures for better water resources management.

Some conclusions can be drawn directly from the reported data, e.g., groundwater storage change reflected by groundwater depth increasing or decreasing. However, in addition to these facts on groundwater storage changes, we also need to determine the dominant drivers behind them, such as the impact of the SNWD on groundwater storage within the context of climate variability and water-related policies, and how these factors may influence groundwater storage in the future. The physical model also provides a solid basis for policy analysis of future water management. Such analyses require integrating the large amount of data into the physically-based hydrologic model. Please also refer to our responses to previous comments.

Comment R2C5: Line 208 “Overall, the central SNWD route, along with suppression of agricultural water use and precipitation variability has reversed the GW decline”: Again, these key features are already clearly seen the data by BWA (Figures 1 and 2). I could not figure out what the authors originally explored through their modeling.

Response to R2C5: Please refer to Response to R2C2 and Response to R2C4. Furthermore, groundwater recovered in different time windows, such as in years 2012‒2013 (Figure 1) prior to initiation of water supply from the SNWD central route. The model is essential for isolating these impacts along with climate variability and/or policies on restrictions in groundwater pumping. In addition, models that accurately reflect the underlying processes are required to develop reliable predictions for the future.

Comment R2C6: Line 357 “Two scenarios for precipitation were formulated … precipitation during 2019-2030 was set to the climatology of precipitation during 2000-2018”: How the sub-annual and inter-annual variations of precipitation were generated? I fully understand near-term climate projection is hard to be carried out, but these would certainly influence the projection of groundwater storage. For insightful discussion, the scenario design is better to be revisited, or at least further elaborated.

Response to R2C6: Initially, precipitation data for the model forcing were derived from the regional climate model (RCM) DMI-HIRHAM5 under RCP4.5 (representative concentration pathway, RCP) within the Coordinated Regional Climate Downscaling Experiment (CORDEX) framework. However, these data were not adopted directly, because of much higher annual magnitudes than those during the observational period, which were not considered realistic for the future. There is also large uncertainty in the inter-annual variability in precipitation from different RCMs and general circulation models (GCMs).

In this study, we focused mainly on the overall trend of precipitation in the future. Thus, we generated precipitation data for future scenarios using the RCM precipitation containing interannual and intra-annual variability, with monthly RCM precipitation corrected to either the climatology (a long-term average) of in situ monthly precipitation during 2000‒2018, resulting in annual precipitation of 540 mm/yr (P1) or to a wet climate during 2008‒2018, resulting in annual precipitation of 580 mm/yr (P2) using multiplicative factors. This is a common way to do for bias correction for precipitation. As a result, short-term variability within a month provided by the RCM was retained, monthly variability was the same as the climatology, but inter-annual variability in precipitation was omitted, to reduce impacts of uncertainties in biased mean annual precipitation and interannual variability on projections of groundwater storage.

To incorporate interannual variability in RCM precipitation in the projections of groundwater storage for future scenarios, we have also conducted another set of projections in response to this review during the revision (Please see Section 3 in the Supporting Information for details). Two new precipitation scenarios during 2019−2030 were generated based on the original RCM precipitation: (P1N) original RCM annual precipitation during 2019−2030 (mean annual precipitation of 750 mm/yr) multiplied by a factor of 0.72 (540/750), to represent the climatology (i.e., mean annual precipitation during 2000−2018, 540 mm/yr), and (P2N) original RCM annual precipitation during 2019−2030 multiplied a factor of 0.77 (580/750), to represent a wet climate (i.e., mean annual precipitation during 2008−2018, 580 mm/yr). Groundwater use scenarios are the same as the initial projections.

New projections of groundwater storage during 2019−2030 clearly show the impacts of interannual variability in precipitation on groundwater storage (Fig.S5 shown below). Increasing trends of the groundwater storage recovery are higher than the initial projections, with the lowest rate of 28 mm/yr under scenario I, compared with 13 mm/yr in the initial projections. However, these trends may have been overestimated, because increased groundwater withdrawal during drought was not incorporated into these scenarios. For instance, annual precipitation in 2026 was estimated to be 282 mm/yr (P1N) after bias correction, which is much less than the historical precipitation level of either 540 mm/yr or 580 mm/yr, and would likely result in substantial groundwater pumping to alleviate the drought. However, the previous finding of overall increasing trends in groundwater storage in Beijing in the coming decade did not change.

We have included this material in the Supporting Information.

Fig. S5| **New projections of GWS change under GW use (normal and low) and precipitation (climatology (P1N) and wet (P2N)) scenarios in Beijing during 2019‒2030**. Same as **Fig.6** in the main text but with new precipitation scenarios (i.e., P1N and P2N).

We have supplemented information on the precipitation scenarios in Lines 386−393.

Technical comments

Comment R2C7: Fig 1 (b,c): Annotations and legends are too small.

Response to R2C7: We adjusted the front size of annotations and legends. Please see the improved Figure 1 in the revised manuscript.

Comment R2C8: Line 98 “Beijing has less than 100 m^3^ per capita water resources”: I guess the number totally depends on the definition of areal extent of city. What is the definition of area for this case (e.g. administrative area or catchment area)?

Response to R2C8: This number was calculated based on the administrative boundary. We have added this information in Line 98.

Comment R2C9: Line 103 “derived from precipitation”. This phrase is unclear and confusing. Both groundwater and surface water are derived from precipitation in a sense.

Response to R2C9: Here we intended to explain that human water use of 2.6 km^3^ was derived from renewable water resources (i.e., surface water and groundwater that is naturally formed by precipitation). We have adjusted the statement in Lines 103−104.

**Reviewer comments, second version:**

Reviewer #1 (Remarks to the Author):

The authors have sufficiently addressed my comments and suggestions for the manuscript and clarified major points of concern. At this point, I feel the manuscript is of a high enough standard and quality, with valuable contributions to the hydrologic community and our understanding of the South-to-North Water Diversion project to be published.


Reviewer #2 (Remarks to the Author):

Dear Authors,

Many thanks for the detailed and informative responses to my earlier comments. I am now basically convinced that all my concerns have been addressed. After reading the response letter, I went through the revised text again and found that some important points in the response letter are not explicitly expressed in body. Please see below for some concrete suggestions. I well acknowledge the authors’ hard work. Hope this manuscript will receive a positive evaluation by this journal.

Abstract: The second sentence introduces South-North Water Diversion (SNWD), and the third sentence (“Here, …”) provides the research objective. I suppose one more sentence should be added between these two to raise the key scientific challenge.

Line 43-56 I’m a bit confused by this paragraph. I cannot contextualize this part (various measures to tackle with droughts in many places in the world including desalination) with this study (develop a hydrological model in the North China Plain to focus on groundwater and water diversion) very easily. I feel this paragraph is unnecessary.

Line 57-59 I feel it is better to merge these lines (information on GRACE) into Line 27-29.

Line 80-94 Now the scientific challenges are clearly defined and the novelty and advances of this study are also concisely presented. Desirably, I would expect a few lines describing the brief structure of forthcoming Results Section. Actually, Results Section start with data analysis, which is not mentioned here at all.

Line 98-198 This is a similar comment to above. This subsection introduces the results of data analyses. Unless carefully guided, readers might be confused because only model simulation was mentioned so far. Eventually, some of the readers may have impression like my original one.

Line 198 For further readability, it would be nice if the authors briefly address what are the unknowns in data analyses and what to be discovered by modeling in the next subsection.

**Author rebuttal, second version:**

Reviewer #1 (Remarks to the Author):

Comment R1C1: The authors have sufficiently addressed my comments and suggestions for the manuscript and clarified major points of concern. At this point, I feel the manuscript is of a high enough standard and quality, with valuable contributions to the hydrologic community and our understanding of the South-to-North Water Diversion project to be published.

Response to R1C1: We deeply appreciate Reviewer #1 for providing the very encouraging evaluation and recognition of this study.

Reviewer #2 (Remarks to the Author):

Comment R2C1:

Dear Authors,

Many thanks for the detailed and informative responses to my earlier comments. I am now basically convinced that all my concerns have been addressed. After reading the response letter, I went through the revised text again and found that some important points in the response letter are not explicitly expressed in body. Please see below for some concrete suggestions. I well acknowledge the authors’ hard work. Hope this manuscript will receive a positive evaluation by this journal.

Response to R2C1: We deeply appreciate Reviewer #2 for these insightful comments and suggestions. We have revised the manuscript accordingly.

Comment R2C2: Abstract: The second sentence introduces South-North Water Diversion (SNWD), and the third sentence (“Here, …”) provides the research objective. I suppose one more sentence should be added between these two to raise the key scientific challenge.

Response to R2C2: We have modified the abstract by adding the sentence that 'impacts of water diversion on GW remained largely unknown' in Lines 15‒16.

Comment R2C3: Line 43-56 I’m a bit confused by this paragraph. I cannot contextualize this part (various measures to tackle with droughts in many places in the world including desalination) with this study (develop a hydrological model in the North China Plain to focus on groundwater and water diversion) very easily. I feel this paragraph is unnecessary.

Response to R2C3: This paragraph aims to put our study in the context of increasing the resilience of water supplies for large cities globally, and to introduce the fundamental factors that may affect groundwater storage changes in terms of inputs/supplies and outputs/demands. Desalination is regarded as a measure to increase input through developing alternative water sources, in addition to water diversion (e.g., what has been implementing in California and Arizona in the US and the South-to-North Water Diversion in China). Therefore, we tend to keep this paragraph in the manuscript, which would also increase the visibility of this study to a broader readership of *Nature Communications*.

Comment R2C4: Line 57-59 I feel it is better to merge these lines (information on GRACE) into Line 27-29.

Response to R2C4: We have improved the expression of the information on GRACE as: 'GRACE satellites monitor changes in Earth’s gravity field that is controlled primarily by variations in total water storage, showing that total water storage is decreasing or getting drier in North China whereas storage is increasing or getting wetter in South China' (Lines 64‒66). This leads to the background of the South-to-North Water Diversion that was designed to transport water from the humid South to the dry North.

Comment R2C5: Line 80-94 Now the scientific challenges are clearly defined and the novelty and advances of this study are also concisely presented. Desirably, I would expect a few lines describing the brief structure of forthcoming Results Section. Actually, Results Section start with data analysis, which is not mentioned here at all.

Response to R2C5: We have added an expression showing a brief structure of the Results Section in Lines 96−98: 'The following Results section shows key analyses and results based on statistical and monitoring data (i.e., the first two subsections) and hydrologic modeling (i.e., the last two subsections).'

Comment R2C6: Line 98-198 This is a similar comment to above. This subsection introduces the results of data analyses. Unless carefully guided, readers might be confused because only model simulation was mentioned so far. Eventually, some of the readers may have impression like my original one.

Response to R2C6: We have shown the structure of the Results section at the end of the Introduction section. As such, the readers might not have such an impression that this study is only based on data analysis. This has also been illustrated in the above response.

Comment R2C7: Line 198 For further readability, it would be nice if the authors briefly address what are the unknowns in data analyses and what to be discovered by modeling in the next subsection.

Response to R2C7: We have provided the motivation of performing the modeling in Line 188: 'To explore the drivers of GWS changes behind the data collected and analyzed above'.

***************************

**Reviewer comments, third version:**

**Author rebuttal, third version:**
